# Exploring Andalusia’s Rich Heritage through Surveys: Pastoral Livestock Farming as a Tourist Attraction Resource

**DOI:** 10.3390/ani14030468

**Published:** 2024-01-31

**Authors:** Francisco de Asís Ruiz Morales, Verónica Cruz Moriana, María Bermúdez Rus, Juan Manuel Mancilla-Leytón, Luis Pablo Ureña Cámara

**Affiliations:** 1 Centro IFAPA Camino de Purchil, 18004 Granada, Spain; franciscoa.ruiz@juntadeandalucia.es (F.d.A.R.M.); veronica.cruz@juntadeandalucia.es (V.C.M.); luispa87@hotmail.com (L.P.U.C.); 2 Máster en Agricultura y Ganadería Ecológicas, Universidad Pablo de Olavide, 41013 Sevilla, Spain; 3 Departamento de Biología Vegetal y Ecología, Facultad de Biología, Universidad de Sevilla, 41012 Sevilla, Spain

**Keywords:** agrotourism, rural tourism, extensive livestock farming, post-COVID era

## Abstract

**Simple Summary:**

The recent health crisis has created a new scenario where rural tourism stands out as a beneficiary of the post-COVID era. The results of this research, based on the conducted surveys, show that agrotourism is an attractive and interesting activity for tourists, offering a diversified experience with livestock-related and pastoral activities in Andalusia (Spain). Agrotourism, a form of rural tourism, has the potential to align with emerging tourist interests, while reactivating the economy of livestock farming families and recognizing the important environmental, social, and economic role of extensive livestock farming. However, based on the findings of this study, specific areas of focus include (i) developing marketing strategies that encourage tourist participation throughout the year; (ii) integrating theoretical and practical content into educational training pro-grams to raise awareness of agrotourism projects and foster interest among future professionals in both the tourism and livestock sectors; and (iii) providing training and support for livestock farmers interested in diversifying their activities through agrotourism (considering such aspects as investment, time commitment, dedication, tourist engagement, forming alliances with companies, etc.). This support could be facilitated by public administrations, fostering synergies between the public and private sectors.

**Abstract:**

This study aimed to analyze the interest, willingness to pay, and profile of tourists participating in specific agrotourism activities linked to extensive livestock farming in order to encourage the development of those activities that are most demanded by tourists, as well as to support the proposals for improvements to promote this kind of tourism in the region of Andalusia (Spain). For this purpose, a survey was conducted, which was organized into six sections: (i) sociodemographic data, (ii) general aspects of tourism, (iii) rural tourism, (iv) livestock farming and tourism, (v) benefits of extensive livestock farming, and (vi) tourism activities linked to livestock farming. A total of 892 responses were received, out of which 753 were analyzed. The results show that the respondents are interested or motivated by the proposed activities. The most attractive activities are those related to livestock farming, which involve some kind of workshop on the production of handicrafts. Despite tourists having low-to-medium knowledge of extensive livestock farming, they all express a highly positive perception of the associated attributes. Developing strategies to enhance the willingness to pay for agrotourism activities is crucial, with the overall experience being a key element of their success.

## 1. Introduction

The traditional tourism model is undergoing a transformation, which includes a growing emphasis on experiential and creative tourism. In the resulting new model, tourists become active participants and protagonists of their own experiences, and tourism is characterized by visitors engaging in educational, emotional, social, and participatory interactions with the destination, its culture, and its local residents [1,2,3,4,5]. The success of the experience lies in authenticity, which is grounded in the diverse characteristics of the territory, including its heritage, landscape, and culture; and the identity of the host population [3,6].

After the COVID-19 pandemic, there is a growing preference for travelling to destinations that primarily emphasize activities and experiences in natural environments [7,8,9]. In response to this demand, rural areas have become one of the most suitable settings. Consequently, ecotourism and rural tourism have emerged in many European countries as two highly favored products [10]. They are developed in open and unspoiled spaces, promoting contact with nature and interaction with the local population in a traditional environment. Moreover, they work to reduce mass tourism, allowing visitors to enjoy the attractions of the place, such as its gastronomy, crafts, sports, cultural activities, etc. Additionally, these forms of tourism contribute to the conservation and maintenance of local resources [5,11,12]. 

As a subtype of rural tourism, agrotourism, defined as “a range of activities, services and amenities provided by famers” [13], presents numerous opportunities for the protection of the rural environment, including the (i) diversification of the family and local economy to counteract rural depopulation; (ii) development of agricultural, livestock, and forestry activities; (iii) attraction of investment through infrastructure improvement; (iv) preservation of the landscape, the ecosystems, and the historical and cultural heritage; and (v) promotion of local products and artisanal forms of production [14,15,16]. 

The Mediterranean Basin and, particularly, Andalusia, Spain’s southernmost region, harbor a rich biodiversity of livestock breeds. The majority of these breeds are found in areas of high environmental value, such as natural parks, national parks, and biosphere reserves. These areas possess significant ethnographic and gastronomic value [17,18]. This activity generates not only marketable and tangible products but also services, known as positive externalities, which help maintain an integral balance between economic, environmental, and social factors [19,20]. For this reason, initiatives should be designed and implemented to enhance the value of heritage resources, and one effective approach to it is through agrotourism [21,22]. Agrotourism facilitates the development of activities related to aesthetic and cultural enjoyment; crafts, industry; leisure and recreation; and other pursuits associated with the breeding, rearing, and grazing of cattle, sheep, goats, and pigs [21,23].

The objective of this study was to analyze the interest, willingness to pay, and profile of tourists participating in agrotourism activities associated with extensive livestock farming in Andalusia (Spain). This analysis, based on surveys, aims to guide the development of agrotourism based on demand, promoting activities that genuinely motivate tourists. Additionally, it seeks to devise new marketing strategies to stimulate public interest in this relatively underdeveloped form of tourism in Southern Europe.

## 2. Materials and Methods

Building on previous studies that explore the status and prospects of agrotourism, along with the attitudes and motivations of tourists towards it [24,25,26], an online survey was designed using the Google Forms tool on the docs.google platform [27], characterized by its multiplatform potential and accessibility.

The survey comprised 29 items organized into three blocks. Block I gathered the respondents’ sociodemographic data, including age, gender, level of education, number of household members, and province of residence. Block II, inspired by the work of Mancilla-Leytón et al. [28], categorized the benefits of extensive livestock farming according to five principles: (i) pastoral livestock farming as a production model that respects animals and the environment; (ii) the prevention of forest fires through pastoral livestock farming; (iii) the conservation of the landscape and territory; (iv) the promotion of biodiversity through the protection of local breeds, fauna and flora, erosion prevention, and soil fertilization; and (v) the generation of products with high sensory and nutritional quality. Finally, in block III, the participants assessed eight tourism activities associated with extensive livestock farming and indicated their willingness to pay for each (<EUR 10; EUR 10–EUR 20; EUR 20–EUR 30; EUR 30–EUR 50; >EUR 50): Activity 1: accompany a shepherd for a full day (transhumance), and enjoy a picnic lunch; Activity 2: get to know the daily life of a dairy farm, put the stock out to pasture, and end the day with a butter-making workshop; Activity 3: visit a fighting-bull farm, and enjoy a snack in the countryside; Activity 4: visit a sheep farm, learn about sheep care, learn how to shear (in spring/summer), and take part in a wool-spinning workshop; Activity 5: go for a walk around a goat farm, milk the goats, visit a cheese factory, and take part in a cheese-making workshop; Activity 6: spend the day with a shepherd, seeing sheep, goats, and/or cows, and enjoy a local breed meat tasting; Activity 7: visit a *dehesa*, learn how pigs are raised in the wild, participate in a pig slaughter, and make your own sausages; and Activity 8: visit a chicken farm, learn about chicken care, collect the eggs laid during the day, and participate in a baking workshop. The questions were presented in a random order to mitigate potential biases associated with question sequence [29]. For data collection, given the online nature of the survey and in order to minimize errors while enhancing participant simplicity [30], a 5-point Likert scale (1 = strongly disagree; 5 = strongly agree) was employed to categorize the statements related to pastoral livestock farming (block II) and tourism activities (block III).

The studied population comprised the whole region of Andalusia, in Southern Spain. Extensive livestock farming has played a fundamental role in this region for millennia, shaping and managing the landscape and serving as a key source of employment for the local population [31]. In addition to its economic contribution, this livestock model provides high-quality food rooted in rural culture that is recognized and sought after by consumers. This dynamic helps maintain a balance between production and the preservation of the ecosystem [32,33]. In contrast, tourism stands out as the economic activity generating the greatest impact in the region.

The data collection process took place in the second half of 2021, utilizing stratified proportional sampling. Maximum quotas of responses were established based on demographic variables such as the respondents’ gender, province of residence, and level of studies to ensure a sample as representative as possible of the Andalusian population [34]. The following exclusion criteria were established: being a minor, residing outside Andalusia, or not participating in tourism and/or rural tourism activities. Additionally, a control question was included throughout the questionnaire to identify and exclude participants who were not paying due attention.

According to the *Instituto Andaluz de Estadística y Cartografía* (Andalusian Institute of Statistics and Cartography) [35,36], the census for 2021 reported a population of 8,371,270, categorized as an infinite population (larger than 100,000 people). To ensure a representative sample with a precision of ±5% at a confidence level of 95.5%, a minimum sample size of 400 respondents was estimated. In this study, a total of 892 surveys were collected. Exclusion criteria were applied to eliminate participants not meeting the specified requirements or providing incorrect answers to the control question. Additionally, straight-liners (respondents consistently providing the same value) were identified, particularly among those with lower levels of education and faster response times [37]. In the end, a total of 753 surveys were included in the analysis.

### Statistical Analysis

The cluster analysis was conducted using the responses obtained for block II (benefits of pastoral livestock farming, 5 variables) and block III (interest in the proposed activities and willingness to pay, 16 variables). The hierarchical cluster analysis was performed using the method of measuring squared Euclidean distances, as well as the nearest-neighbor method for the elimination of outliers [38]. Subsequently, Ward’s method was applied to identify resulting groups based on the analysis of 21 variables. These groups were further validated through one-way ANOVA comparisons. A factor analysis was then conducted, including a Kaiser–Meyer–Olkin (KMO) test to assess the appropriateness of applying this reduction, and Bartlett’s test of sphericity. Finally, a principal component extraction was carried out, selecting variables with eigenvalues for total variance explained exceeding 1. The Pearson test was conducted to measure the strength and direction of the linear relationship among the study variables. The statistical analysis was performed using IBM SPSS Statistics for Windows v 26 (IBM Corp. Released 2017 Armonk, NY, USA).

## 3. Results

The distribution of the surveys analyzed closely resembled that of the Andalusian population in terms of gender, age, province, and household size (Table 1). However, there was a distortion in the distribution by educational levels, with a tendency towards higher education. Additionally, the over-65 age group had the lowest participation rates in relation to the population of Andalusia (Table 1).

The analysis of the components involved examining the loadings and contributions of each variable to the identified factors, providing insights into the underlying structure of the data. Based on the KMO test and Bartlett’s sphericity test, four components were extracted, which explained 67.41% of the total variance (KMO = 0.828, gl = 210, *p* ≤ 0.05; Table 2). 

Based on the aforementioned results, the 753 surveys were categorized into four segments or clusters. (i) Cluster 1 is individuals motivated by extensive livestock farming (*n* = 208, 27.6%). These are the consumers who most highly value the attributes associated with pastoralism, demonstrating greater interest and a willingness to pay more for each of the proposed activities than other groups (Table 2). (ii) Cluster 2 consists of individuals interested in extensive livestock farming (*n* = 316, 42%). They are eager to participate in tourism activities related to livestock but are not willing to pay a significant amount for them (Table 2). (iii) Cluster 3 contains individuals interested in product processing workshops (*n* = 121, 16.1%). They have limited knowledge about pastoral livestock farming, but they hold a positive regard for it and are highly interested and willing to pay for activities related to ruminants (Table 2). (iv) Cluster 4 consists of individuals who are indifferent to extensive livestock farming (*n* = 108; 14.3%). Despite their positive appraisal of the attributes associated with pastoralism, these consumers exhibit a significant lack of interest in engaging in related activities and provide very low willingness-to-pay scores (Table 2).

Table 3 presents the results of the sociodemographic variables (gender, age, and level of education) for each cluster. Overall, the survey conducted for this study achieved a well-balanced participation off both men and women (men = 51.3%; woman = 48.7%). While in Clusters 1 and 2, men and women were almost equally represented, differences were observed in Clusters 3 and 4. In Cluster 3, the proportion of men (78.5%) was much higher than that of women (21.5%), whereas in Cluster 4, women constituted the majority (60.2%). Nevertheless, gender does not exhibit a significant correlation with the identified clusters (*p* ≥ 0.05). In terms of age, the more prominent age ranges were 36–50 (33.7%) and 51–65 (33.1%), followed by 18–35 (26.2%). In Clusters 1 and 2, participation across the 18–35, 36–50, and 51–65 age groups remained constant, but differences reappeared in Clusters 3 and 4. In Cluster 3, the 36–50 age range increased (44.6%), while in Cluster 4, the increment occurred in the 51–65 age group (47.2%). In all clusters, the over-65 age range exhibited the lowest participation rates (3.3–7%). The correlation between age and clusters was found to be statistically significant, albeit the correlation coefficient was low (R^2^ = 0.016; *p* ≤ 0.05). In terms of educational level, the majority of participants had completed higher education (84.5%), while the proportion of participants with secondary and primary education was minimal (13% and 2.5%, respectively). Participation was consistent across the clusters, except in Cluster 3, where no participants had primary education. Finally, the respondents’ knowledge of livestock farming was predominantly low to medium (27.8% to 47.4%). The differences between clusters were minimal, except when compared with Cluster 1, which exhibited the highest level of knowledge about livestock farming (Table 3). In any case, there was no statistically significant correlation between both variables in this study (*p* ≤ 0.05).

The environmental attributes associated with pastoralism exhibited very high mean values (Table 3): the generation of products of high sensor and nutritional quality, fire prevention, and environment protection were the three best-rated attributes (4.67, 4.65, and 4.62 out of 5, respectively). The scores obtained for each cluster follow a consistent pattern, with Cluster 1 assigning significantly higher scores and Cluster 4 assigning the lowest scores to these attributes (*p* ≤ 0.001). 

With regard to interest shown in each of the activities linked to pastoral livestock farming, the present study yields general results that point in the same direction, with consumers displaying a high level of interest and more than half of the activities (63%) receiving a score above 4 out of 5 (Table 3). The activity with the highest overall score was Activity 5 (visit a goat farm and a cheese factory and take part in a cheese-making workshop; score 4.25 out of 5), while that with the lowest scores was Activity 8 (visit a chicken farm and participate in a baking workshop; score 3.54 out of 5). As for the respondents’ interest, for all evaluated activities, Cluster 1 significantly gave the highest score, while Cluster 4 ranked the lowest (*p* ≤ 0.001); and intermediate scores were observed in Clusters 2 and 3. The remaining activities had intermediate scores (Table 3). 

Despite the high scores of interest for the proposed activities, the average willingness to pay was low (EUR 10–EUR 20). The activities where the highest willingness to pay was highest are associated with spending the day with a shepherd, cheese making, and transhumance (Activities 6, 5, and 1). Visiting a chicken farm and participating in a baking workshop (Activity 8) exhibited the lowest willingness to pay, while the remaining activities reached intermediate values (Table 3). This pattern persisted across all activities, with Cluster 1 showing significantly higher willingness-to-pay values and Cluster 4 displaying the lowest values (EUR 20–EUR 30 and <EUR 10, respectively, *p* ≤ 0.001), while intermediate values were observed in Clusters 2 and 3 (EUR 10–EUR 20). Interest in the proposed activities and willingness to pay were significantly correlated (R^2^ = 0.84; *p* ≤ 0.05).

## 4. Discussion

One of the reasons to quantify the growth of agritourism in many European countries is that relatively few countries collect precise statistics [6]. The limited number of research studies carried out in Spain runs in parallel with the limited development of agrotourism, thus making it challenging to analyze such factors as the tourists’ interest and willingness to pay and the specific profile of those eager to participate in agrotourism activities associated with extensive livestock farming [24]. Most of the existing research has focused on the impact of tourism activities on traditional practices such as livestock farming [20,25,26]. However, agrotourism is a burgeoning economic activity, social practice, and industry [39], as evidenced by the results of various surveys where respondents demonstrated significant interest in rural tourism and agrotourism activities [25]. Despite this growing interest, there are still few companies engaged in this kind of activity, and livestock farmers remain hesitant due to the lack of knowledge and time constraints. And yet, initiatives are already being developed by public administrations and various companies.

According to data gathered from the *Observatorio de Turismo Rural* (Spanish Rural Tourism Observatory) [40] for year 2022, the typical profile of a rural traveler is that of a woman aged 40 to 64 who seeks a destination offering tranquility and a connection with nature, while also providing active tourism options. The survey conducted for this study achieved a balanced participation of both men and women. In Cluster 1, the participation of men is slightly higher than that of women (51.4% and 48.6%, respectively), with a relatively homogeneous distribution across age groups, except for the over-65 age group, where the difference between gender groups is notably higher. Despite the low percentage of livestock knowledge (40.4%), this cluster shows the highest appreciation for attributes linked to pastoralism. In Cluster 2, women constitute a slightly larger share than men (55.4% and 44.6%, respectively), with majorities in the 36–50 and 51–65 age groups. While knowledge about livestock farming is low, this cluster also holds positive values for attributes linked to pastoralism. Cluster 3 is notable for having the highest representation in the 36–50 age range (44.6%). Men dominate this cluster (78.5%). With regard to knowledge of livestock farming, it registers high values for the “low” category and 0 for “very high”. Nevertheless, members of this cluster exhibit a very positive valuation of pastoralism and its attributes. Lastly, in Cluster 4, women are the majority (60.2%), with the 51–65 age group representing the highest percentage (47.2%). Notably, this cluster showed an increase in the “no knowledge” category of the variable “knowledge about livestock farming”, even if the attributes linked to pastoralism were positively valued. It is worth mentioning that, across all clusters, respondents tended to have high educational levels, ranging from 81.5% to 90.9%. This skew may be attributed to the greater access to online survey tools among individuals with higher education [41] and to the process of eliminating straight-liners. However, the distribution of the surveys studied was very similar in terms of gender, age, province, and household size to that of the Andalusian reference population, so that it can be affirmed that the results obtained have a good representativeness.

According to the research conducted by Ruiz Morales et al. [42], livestock farming is primarily associated with nature tourism (75%). Likewise, as outlined in the study by Leco et al. [25], rural tourists demonstrate environmental awareness, with 92% of them considering that agrotourism contributes to preserving the landscape of farms. In the present study, the environmental attributes associated with pastoralism display notably high mean values: 4.62 for environment protection, 4.65 for fire prevention, 4.58 for landscape and land conservation, and 4.60 for promotion of biodiversity. Another externality associated with extensive livestock farming is the output of high-quality products, which have become integral elements of territorial and heritage dynamism in rural areas. In this study, the statement “pastoral livestock farming generates products of high sensory and nutritional quality” was highly valued, earning the highest score (4.67 out of 5).

Agrotourism activities have evolved beyond merely providing accommodation services, which was the initial focus. Instead, they are now closely tied to the farm and to agrifood production, including activities such as farm visits, product tastings, and sales [43]. Additionally, they involve the interpretation and/or understanding of livestock heritage [44,45]. According to the study conducted by Ruiz Morales et al. [46], 60% of the respondents considered local pastoral sheep farming with meat aptitude (Segureña breed) as a complementary resource to other tourist activities, and 20% viewed it as an activity with great potential on its own. In Ruiz Morales et al.’s study [47], for the local Murciano-Granadina dairy goat breed, the percentage reached 46.2% for both alternatives. All the activities proposed in this research are aligned with this emerging agrotourism trend, where animal husbandry, farm management, agrifood products, and, notably, the visitor’s experience and emotional interaction through participation in workshops play a central role. In terms of the expressed interest in activities associated with pastoral livestock farming, the current study presents positive results, indicating a high level of interest among consumers (most of the activities received a score of more than 4 out of 5). Consistent with the results obtained, the highest-rated activities were Activity 5 (visit a goat farm and a cheese factory and take part in a cheese-making workshop, 4.25 out of 5), Activity 1 (transhumance, score 4.18 out of 5), Activity 4 (visit a sheep farm and participate in a wool-spinning workshop, score 4.06 out of 5), and Activity 6 (spend a day with a goat, sheep, and/or cow herder and participate in a local breed meat tasting, score 4.03 out of 5).

Among animal products, cheese has notably developed a link to tourism, with examples across various species: goat, sheep, cow, and even buffalo [48,49,50,51]. As for their willingness to pay, given the general inclination not to rate any activity very highly, it is worth noting that, on average, rural tourists tend to spend less money on the activities than other tourists [40]. In Spain, there are areas where cheese and dairy products, such as the Cabrales cheese PDO [52] or El Roncal cheese PDO [53], are already utilized as tourist resources. Traditional productions or products under Protected Designations of Origin (PDOs) or Protected Geographical Indications (PGIs) also contribute significantly to the tourist development of an area. These designations certify the inherent connection of those products with the territory, encompassing local practices, knowledge, and management systems rooted in a longstanding tradition [54,55]. In this sense, food has become part of human heritage [56], transforming into a tourist resource that significantly influences the choice of tourist destinations [57,58,59]. In the particular case of cheese, a significant connection between tourism and the processing sector has been established, with innumerable examples found worldwide [60,61]. Similarly, there are models that integrate tourism with various meat products, as seen in the example of Iberian ham [62,63].

Market segmentation has encouraged the creation of new tourism products centered on gastronomy, where experiences and sensations become especially relevant [64]. Activity 6 (spend a day with a goat, sheep, and/or cow herder and participate in a local breed meat tasting) ranked fourth in average score and attained the highest average score in willingness to pay. Local gastronomy emerges as a tourist resource due to its authenticity, and the presence of local breeds contributing raw materials to it should play a crucial role in many rural areas [65]. The same applies to the use of wool; while the use of wool has experienced a decline in recent decades, its natural origin and textile properties have led to a revival in the fashion industry [66]. The utilization of wool (Activity 4) as a tourist resource is still budding, but from its procurement (shearing of sheep) and refining to the spinning process and its incorporation into textiles, it is progressively becoming an activity of interest to tourists [67].

The second highest-rated activity was Activity 1 (Transhumance). Despite being categorized as an Intangible Cultural Heritage of Humanity by UNESCO [68], this activity only secured the second-highest average score in the overall research (4.18) and the third-highest willingness to pay (2.70). Despite the decrease in transhumance as a livestock management practice in recent years [69], its appeal as a tourist activity has risen [70,71], making its valorization a potential option to promote its continued existence [72]. In contrast with Activity 1, the interest scores for Activity 3 (visit a fighting-bull farm and enjoy a snack in the countryside) and Activity 7 (visit a *dehesa*, participate in a pig slaughter, and learn to make sausages) were relatively low (3.69 and 3.61 out of 5, respectively). This contrasts with the findings of Sayadi et al. [24], who reported that tourists positively appreciated both activities. Despite the obtained scores, all activities developed around the Iberian pig, including tastings, fairs, slaughters, etc., as well as the experience of visiting a *dehesa* and learning about extensive breeding, are tourist resources positively valued in other studies [62,63]. This may be due to the fact that national and foreign tourists appreciate and are interested in the autochthonous ecosystems and customs of Andalusia much more than its own inhabitants are [73]. As regards bulls, bullfighting can no longer be the sole activity generating economic returns from their breeding. It is necessary to design an attractive tourist offer, encompassing everything from breeding to commercialization [74]. While the fighting bull is a hallmark of Spanish culture, new approaches must be sought to enhance its value [75], considering its genetic resources, unique management practices, and the agroecosystem of the pastures where they are bred [76].

It has become evident that the attributes of pastoral livestock farming in Andalusia have the potential to drive rural development by linking agrotourism activities to active and nature tourism, as well as gastronomic tourism and other economic endeavors within rural areas. However, based on the findings of this study, specific areas of focus include (i) developing marketing strategies that encourage tourist participation throughout the year; (ii) integrating theoretical and practical content into educational training programs to raise awareness of agrotourism projects and foster interest among future professionals in both the tourism and livestock sectors; and (iii) providing training and support for livestock farmers interested in diversifying their activities through agrotourism (considering such aspects as investment, time commitment, dedication, tourist engagement, forming alliances with companies, etc.). This support could be facilitated by public administrations, fostering synergies between the public and private sectors.

## 5. Limitations and Future Research

Some of the limitations identified in this study may suggest future lines of research, including (i) expanding the sample to achieve greater representativeness of the over-65 age group and individuals with primary and secondary education levels; (ii) extending the study’s boundaries to include potential tourists from other regions, such as Extremadura, Madrid, Valencia, or Catalonia [46]; (iii) assessing the environmental functions associated with grazing in monetary terms to establish the costs of the activities to be carried out; (iv) incorporating other socioeconomic and cultural aspects that are specific to different areas within Andalusia (agricultural practices, the effect of PDOs and PGIs, protected natural areas, costumes, culinary traditions, etc.); and (v) digitizing the livestock sector to obtain resources that can be used in the promotion and development of tourist activities. This approach should consider seasonal variations, diverse resources, and the unique appeal of each season.

## 6. Conclusions

The results of this research show that agrotourism is an attractive and interesting activity for tourists, offering a diversified experience with livestock-related and pastoral activities. Concurrently, this practice serves as a stimulus for the local economy, underscoring the crucial role of extensive livestock farming in environmental, social, and economic aspects. Despite a moderate level of knowledge about extensive livestock farming, the surveyed individuals exhibit an awareness of its benefits and hold positive perceptions of its environmental attributes, as well as of the production of high-quality food associated with it.

Although the respondents’ willingness to pay was not very high, a significant number expressed interest in and motivation for all proposed activities, particularly those associated with shepherding that include artisanal production workshops. The experiential aspect emerges as a crucial element for the development of agrotourism. Participating in transhumance, experiencing the daily life of a shepherd, joining a cheese workshop, and learning to spin or make sausages proved their attractiveness and their alignment with the evolving interests of contemporary tourists.

Further research will be essential to comprehensively analyze the tourists’ profile and the supply and demand of agrotourism activities, which is crucial to respond to the emerging interests and to recognize the pivotal role of extensive livestock farming in meeting the demands of tourists.

## Figures and Tables

**Table 1:** Main descriptors (%) of the surveys carried out (*n* = 753) in contrast to the officially published records of Andalusia (Spain).

	Study Sample	Andalusia ^1^
**Gender**		
Male	48.74	49.33
Female	51.26	50.66
**Place of residence (province)**		
Almería	8.23	8.63
Cádiz	15.01	14.71
Córdoba	9.96	9.17
Granada	9.30	10.88
Huelva	6.90	6.20
Jaén	7.30	7.40
Málaga	19.52	20.01
Sevilla	23.77	22.99
**Age (years)**		
18–35	26.16	25.38
36–50	33.73	29.39
51–65	33.07	25.10
>65	7.04	20.13
**Educational level**		
Primary	2.52	26.10
Secondary	13.01	47.10
Higher	84.46	26.80
**Numbers of residents in the home**		
1	11.81	24.26
2	26.56	28.37
3	19.78	21.01
4	27.75	19.85
≥5	14.07	6.48

^1^ Andalusia Institute of Statistical and Cartography [35,36].

**Table 2:** Matrix evaluating data adequacy for structure detection based on the Kaiser–Meyer–Olkin (KMO) test and Bartlett’s test.

Study Variables	Component 1	Component 2	Component 3	Component 4
Willingness to pay “Sheep farm + wool-spinning workshop”	0.853			
Willingness to pay “Goat farm + cheese-making workshop”	0.844			
Willingness to pay “Cow farm + butter-making workshop”	0.802			
Willingness to pay “Transhumance + picnic lunch”	0.765			
Willingness to pay “Chicken farm + baking workshop”	0.727			
Willingness to pay “Sheep/goat/cow + meat tasting”	0.703			0.434
Interest “Sheep farm + wool-spinning workshop”		0.823		
Interest “Goat farm + cheese-making workshop”		0.794		
Interest “Cow farm + butter-making workshop”		0.784		
Interest “Chicken farm + bakery”		0.741		
Interest “Transhumance + picnic lunch”		0.643		
Interest “Sheep/goat/cows + meat tasting”		0.572		0.538
Grazing preserves the landscape and the territory			0.867	
Grazing promotes biodiversity			0.845	
Environmentally and animal-friendly production system			0.810	
Grazing prevents forest fires			0.792	
Grazing generates products of high sensory and nutritional quality			0.687	
Interest “Iberian pig farm + sausages”		0.336		0.760
Interest “Fighting-bull farm + snack”				0.756
Willingness to pay “Iberian pig farm + sausages”	0.492			0.676
Willingness to pay “Fighting-bull farm + snack”	0.472			0.644

**Table 3:** Categorization of the study population (gender, age, level of education, and livestock knowledge), assessment of different attributes linked to livestock farming, and interest and willingness to pay for the tourist activities proposed for each cluster.

	Total (*n* = 753)	Cluster 1 (*n* = 208)	Cluster 2 (*n* = 316)	Cluster 3 (*n* = 121)	Cluster 4 (*n* = 108)	Statistical Value
**Social variables (%)**						
Gender						
Male	51.3	51.4	44.6	78.5	39.8	--
Female	48.7	48.6	55.4	21.5	60.2	--
Age (years)						
18–35	26.2	27.9	28.5	28.1	13.9	--
36–50	33.7	34.1	33.2	44.6	22.2	--
51–65	33.1	31.3	32.9	24.0	47.2	--
>65	7.0	6.7	5.4	3.3	16.7	--
Educational level						
Primary	2.5	3.8	2.5	0.0	2.8	--
Secondary	13.0	13.0	13.6	9.1	15.7	--
Higher	84.5	83.2	83.9	90.9	81.5	--
Livestock knowledge						
None	13.0	8.2	13.9	14.0	18.5	--
Low	47.4	40.4	47.5	54.5	52.8	--
Medium	27.8	31.3	28.5	24.0	23.1	--
High	8.9	14.9	7.3	7.4	3.7	--
Very high	2.9	5.3	2.8	0.0	1.9	--
**Assessment of attributes related to extensive livestock farming ^1^**						
Environmentally and animal-friendly production system	4.62	4.83 ^a^	4.60 ^b^	4.47 ^b^	4.46 ^b^	10.76
Grazing prevents forest fires	4.65	4.85 ^a^	4.59 ^b^	4.60 ^b^	4.48 ^b^	8.20
Grazing preserves the landscape and the territory	4.58	4.78 ^a^	4.53 ^b^	4.52 ^b^	4.44 ^b^	7.27
Grazing promotes biodiversity	4.60	4.83 ^a^	4.53 ^b^	4.59 ^b^	4.38 ^b^	11.61
Grazing generates products of high sensory and nutritional quality	4.67	4.86 ^a^	4.69 ^b^	4.53 ^c^	4.43 ^c^	14.15
**Interest in proposed activities ^2^ and willingness to pay ^3^**						
Act. 1. Transhumance and enjoy a picnic lunch						
Interest	4.18	4.79 ^a^	4.28 ^b^	4.16 ^b^	2.72 ^c^	124.71
Willingness to pay	2.70	3.57 ^a^	2.54 ^b^	2.46 ^b^	1.73 ^c^	82.61
Act. 2. Visit a cow farm and participate in a butter-making workshop						
Interest	4.02	4.68 ^a^	4.20 ^b^	4.15 ^b^	2.05 ^c^	237.28
Willingness to pay	2.46	3.34 ^a^	2.26 ^b^	2.48 ^b^	1.35 ^c^	104.44
Act. 3. Visit a fighting-bull farm and enjoy a snack in the countryside						
Interest	3.69	4.41 ^a^	3.81 ^b^	2.88 ^c^	2.86 ^c^	57.58
Willingness to pay	2.35	3.29 ^a^	2.18 ^b^	1.55 ^c^	1.94 ^bc^	70.23
Act. 4. Visit a sheep farm and participate in a wool-spinning workshop						
Interest	4.06	4.76 ^a^	4.11 ^b^	4.30 ^b^	2.32 ^c^	205.19
Willingness to pay	2.58	3.50 ^a^	2.31 ^c^	2.61 ^b^	1.54 ^d^	114.31
Act. 5. Visit a goat farm and cheese factory, and participate in a cheese-making workshop						
Interest	4.25	4.86 ^a^	4.29 ^c^	4.53 ^b^	2.65 ^d^	183.81
Willingness to pay	2.71	3.59 ^a^	2.42 ^c^	2.77 ^b^	1.81 ^d^	101.14
Act. 6. Spend the day with a shepherd, seeing sheep, goats, and/or cows, and enjoy a local breed meat tasting						
Interest	4.03	4.83 ^a^	4.33 ^b^	3.28 ^c^	2.46 ^d^	201.69
Willingness to pay	2.72	3.72 ^a^	2.74 ^b^	1.80 ^c^	1.80 ^c^	116.41
Act. 7. Visit a *dehesa*, participate in a pig slaughter, and make your own sausages						
Interest	3.61	4.47 ^a^	4.24 ^a^	1.65 ^bc^	2.31 ^b^	301.48
Willingness to pay	2.33	3.56 ^a^	2.60 ^b^	0.35 ^d^	1.43 ^c^	316.97
Act. 8. Visit a chicken farm and participate in a baking workshop						
Interest	3.54	4.37 ^a^	3.41 ^c^	3.85 ^b^	1.99 ^d^	116.05
Willingness to pay	2.06	3.08 ^a^	1.70 ^c^	2.07 ^b^	1.11 ^d^	118.38

**^1^** 1: Strongly disagree; 2: disagree; 3: neither agree nor disagree; 4: agree and 5: strongly agree. **^2^** 1: Not interesting; 2: a little interesting; 3: neutral; 4: interesting and 5: very interesting. **^3^** 1: <EUR 10; 2: EUR 10–EUR 20; 3: EUR 20–EUR 30; 4: EUR 30–EUR 50; 5: >EUR 50. ^a–d^ Values with different letters on the same row mean significant difference (*p* ≤ 0.001).

## Data Availability

Data are contained within the article.
